# Oral and dental lesions in HIV infected Nigerian children

**DOI:** 10.11604/pamj.2015.20.287.5273

**Published:** 2015-03-24

**Authors:** Olusola Adetunji Oyedeji, Olalere Omoyosola Gbolahan, Elizabeth Oluwatoyin Abe, Efeturi Agelebe

**Affiliations:** 1 Department of Paediatrics, Ladoke Akintola University of Technology Teaching Hospital, Osogbo, Osun State, Nigeria; 2 Department of Oral and Maxillofacial Surgery, University college Hospital, Ibadan, Oyo State, Nigeria; 3 Department of Oral Pathology, University college Hospital, Ibadan, Oyo State, Nigeria

**Keywords:** Paediatric, oral, disease and HIV

## Abstract

**Introduction:**

Oral diseases in the HIV infected children though commonly encountered are under researched and often overlooked by physicians in developing countries. The aim of this study is to document the types and frequency of oral lesions in HIV infected children and examine the effects of management with HAART on their rates.

**Methods:**

A cross sectional study designed to identify the oral lesions in consecutive HIV infected children and their distribution at a Paediatric Anti-retroviral clinic. Information on oral disease and clinical features of the subjects were obtained by history and clinical examination and laboratory investigations by the pediatricians and dental surgeons.

**Results:**

The 58 children studied consisted of 34 boys and 24 girls with their ages ranging from 3 months to 13 years. Thirty seven (63.8%) of the 58 children had oral diseases. Enamel hypoplasia, candidiasis, caries, angular chelitis, and herpes labialis were the most common oral lesions found in the patients. Oral soft tissue lesions were less frequently encountered among children on HAART. Statistical significance was recorded among those infected with candidiasis. More than 60% of the children diagnosed with oral disease had no knowledge of the state of their oral health before the study.

**Conclusion:**

Oral diseases are very common amongst the children studied. Awareness of oral disease among the children and their caregivers is low. Administration of HAART may have a preventive effect on the development of oral soft tissue disease. There is a need to integrate dental care into the paediatric HIV care programs.

## Introduction

Human immunodeficiency virus infection continues to be a significant cause of morbidity and mortality. Efforts aimed at controlling the disease seem inadequate considering a recent report stating a 2.5million incidence of new infections globally with Sub-Saharan Africa accounted for 70% of the infections [1]. Nigeria is the most populous country in sub-sahara Africa and it has one of the highest burden of paediatric HIV globally [1]. Paediatric HIV Infection is a problem because of the multi-systemic disease affectation, faster disease progression and severity due to the immaturity of the child's immune system. The multi-systemic affectation of HIV exerts a negative impact on the quality of life of the infected by causing malfunction of several body systems. A benefit in disguise of the affectation of multiple systems is that it makes detection and tracking of the disease easier. Human immunodeficiency virus has significant oral manifestations and certain lesions may signify the earliest clinical signs of infection and progression [2]. Oral manifestation of candidiasis and oral hairy leukoplakia in particular are clinical predictors of Acquired Immune Deficiency Syndrome progression and their presence usually indicates a CD4 count below 200cells/mm^3^ or a very high viral load [3, 4] This study aims to determine the pattern and frequency of oral lesions in HIV infected children in addition to comparing the rates of oral lesions among those on HAART and those not on HAART. This study also seeks to do a situational analysis of oral health practices and suggest what can be done to improve on the existing practices.

## Methods

Consecutive HIV infected children attending the paediatric Anti-retoviral (ARV) clinic of the Ladoke Akintola University Teaching Hospital, (LAUTECH), Osogbo between 1^st^ of July and December 31^st^ 2013 were studied. Ethical approval was obtained from the ethics and research committee of LAUTECH, Osogbo. Informed consent was obtained from the parents of the children studied. Diagnosis of HIV in children older than 18months was based on testing positive to the ELISA (HIV) test and further confirmation using the western blot technique. Children aged between 6 weeks and 18 months were diagnosed to be HIV infected when their blood samples tested positive to the polymerase chain reaction and this was further confirmed by repeat tests. Subjects were seen by the paediatrician in charge of the paediatric ARV and given routine care at the Paediatric ARV clinic of LAUTECH, Osogbo. Information concerning the state of health and complaints of the patients were obtained after which the patients received a comprehensive examination to determine their WHO clinical stages and fitness there after care was assessment based. The tests routinely done at the ARV clinic include complete blood count and CD4 counts. Highly Active Antiretroviral therapy and Co-trimoxazole were also prescribed, according to the National guidelines [5]. The oral cavity and the surrounding structures were also examined by two trained dental surgeons on completion of assessment and consultation with the paediatrician at the same location. Enquiries were made concerning dental complaints, dental hygiene practice and the consumption of refined carbohydrates or sugar. There after the oral cavity was examined and the details on the finding were recorded in the proforma specially designed for the study. Further education and counselling was provided on oral health on completion of their assessment. Additional care for oral pathologies was provided by prescriptions or referrals were appropriate, since the teaching hospital of study lacks a dental center. Data was entered into a computer and analyzed using SPSS version 19. Associations were determined for categorical variables and expressed as ratios and percentages. Significance of the associations was based on tests such as P value and odds ratio using the 95% confidence interval. The means, standard deviation and ranges of continous variables were also computed and used to express the results.

## Results

### The subjects studied

Fifty eight children were seen over the six months study period and this accounts for 92.1% of the total 63 children enrolled in the Paediatric Antiretroviral clinic at the time of the study. The age of the children studied ranged from 3 months to 13 years, while the mean(S.D) of their ages was 5.7(+/_ 3.1). Thirty four of the children studied were boys and 24 were girls, giving a male to female ratio of 1:0.7.

### Prevalence and pattern of oral disease

Of the 58 children studied, 37 (63.8%) had oral diseases while 21 (36.2) did not. Ten of the 37 patients with oral disease had at least two oral diseases, while another 2 had 3 oral diseases. The common oral diseases reported amongst the children studied include enamel hypoplasia, oral candidiasis and caries. Thirty seven children had enlargement of the regional lymph nodes draining the oral cavity. All the thirty seven had submandibular lymph node enlargement, while two of these children also had associated enlargement of either the sublingual or cervical lymph nodes. Table 1 shows the pattern of oral diseases seen.

**Table 1:** Socio-demographic details, WHO staging, mouth cleaning, and sugar ingestion by the 58 HIV infected children

Variable	Number (n = 58)	Percentage
**Age category**		
0 - 5	26	44.8
>5 - 10	27	46.6
>10	5	8.6
Gender		
Male	34	54.6
Female	24	41.4
**Social class**		
I	3	5.2
II	15	25.9
III	14	24.1
IV	25	43.1
V	1	1.7
**WHO HIV STAGING**		
I	4	6.9
II	29	50.0
III	23	39.7
IV	2	3.4
**Mouth cleaning**		
Yes	53	91.7
No	5	8.3
**Regularity of mouth cleaning**		
Once a day	49	84.5
More than once a day	5	8.6
Less than once a day	1	3.4
Nil /Not applicable	3	5.2
**Device used in tooth cleaning**		
Tooth brush and tooth paste	47	78.3
Cotton wool and tooth paste	3	5.0
Foam and tooth paste	3	5.0
Chewing stick	1	1.7
Salt and water and cotton wool	1	1.7
**Consumption of sugary snacks**		
Once a day	10	16.7
> Once a day	25	41.7
Occasionally	14	23.3
Abstinence from sugary snacks	11	18.3

### Age and sex distribution of the children with oral diseases

The rates of oral diseases amongst both sexes were similar with 21(61.8%) of the 34 boys recording oral diseases and 16(66.7%) of the corresponding 24 girls also recording similar oral diseases. The differences recorded across the sexes are not statistically significant. X^2^ = 0.15, p = 0.70). The details of these associations are shown in Table 2

**Table 2:** The oral diseases diagnosed in the subjects

Oral disease	Number (n = 58)	Percentage of 58
Enamel hypoplasia	26	44.8
Oral candidiasis’	10	17.2
Caries	7	12.1
Chelitis	5	8.6
Herpes labialis	4	6.8
Linear gingiva erythma	3	5.2
Gingivitis	2	3.4
Parotid gland enlargement	2	3.4
Submandibular gland enlargement	2	3.4
Apthous ulcers	1	1.7

### Oral care

Cleaning the mouth was by use of tooth brush with toothpaste in 47(78.3%) participants, cotton wool or foam with toothpaste in 6(10.0%) participants while 5 participants (3 infants yet to erupt teeth and 2 older dentate children) did not use anything to clean their mouth. Frequency of mouth cleaning was at least once a day in 53 and nil in the remaining five.

### Presenting complaints

Twelve caregivers (20.7%) had complaints at presentation and 46(79.3%) had none. Thus 25(67.6%) of the 37 children diagnosed to have oral disease in the course of this study, gave no complaints at presentation. These 25 children or caregivers hitherto unaware of their oral disease constitute 43.1% of the 58 studied children. Of the 58 children, 4(6.9%) complained of a white discoloration of the tongue or buccal mucosa while another four (6.9%) complained of brownish yellow discoloration of the teeth. The remaining four children complained of different problems. The complaints were unduly mobile tooth, tooth aches, tooth decay and pains on chewing.

### Prior medications

Of the 12 children that had presenting complaints, 6(50.0%) were using some form of medications prior to visiting a health facility. Four of the patients did not know the names of the drugs they were taking. Of the remaining two children one of the children had salt and water administered by cotton wool to the mouth and the other took an unknown concoction.

### Previous dental visits

All the children studied had never visited a dental facility or a dentist for check up or treatment. Of the 58 children 54(93.1%) gave reasons of not having a dental problem necessitating visit, while another 2(3.4%) stated that their children were still infants and yet to get to the stage of tooth eruption and the 2(3.4%) remaining gave reasons of ignorance of the need for a dental check as reasons for failure to visit a dentist for a check up hitherto.

### WHO clinical staging and Immune status

According to the WHO clinical staging for HIV 4(6.9%), 29(50.0%), 23(39.7%) and 2(3.4%) had stage I, II, III and IV disease clinical staging respectively. The mean CD4 (S.D) of the children studied was 759.87cells/mm^3^. Three children had a CD4 below 200cells/mm^3^ while 17 of them had a CD4 below 15%. There was no significant association between the numbers of children with oral diseases at CD4 values > 350 cell/mm^3^ and below for children aged 5 years and above. Similarly, no significant association was recorded for oral diseases at CD4 counts > 20% and below the expected for age for children under the age of 5 years. The association between dental and oral lesions, WHO clinical staging and immune status is depicted in Table 2.

### Association between the use of HAART and oral disease

Of the 58 children, 37 had initiated HAART and 21 were yet to be initiated on HAART. Twenty three(62.2%) of the 37 children with oral diseases were on Highly Active Anti-retroviral Therapy and 14(37.8%) were not, compared with the 17(81.0%) children with oral diseases amongst the 21 who were not on HAART. These differences are not statistically significant. (P = 0.14, OR = 0.39, 95% CI = 0.11 - 1.39). The association between the different oral diseases and the use of HAART is shown in Table 3.

**Table 3:** Distribution of soft oral tissue and dental diseases according to the age, sex, social class, WHO clinical staging and immune status of the studied children

Variable	Number (%) with Oral diseas (n = 37)	Number (%) without oral disease (n =21)	P Value	Odds ratio	95% confidence interval
**Age**					
0 - 5	14 (53.8%)	12(46.2%)	0.13		
>5 - 10	18(66.7%)	9(33.3%)			
>10	5(100.0%)	0(0.0%)			
**Sex**					
Male	21(61.8%)	16(38.2%)	0.70	0.81	0.27 - 2.41
Female	13(66.7%)	8(33.3%)			
**Social class**					
I	2(33.3%)	7(66.7%)	0.15		
II	8(33.3%)	7(46.7%)			
III	6(42.9%)	8(57.1%)			
IV	20(80.0%)	5(20.0%)			
V	1(100.0%)	0(0.0%)			
**WHO clinical stage**					
I	2(50.0%)	2(50.0%)	0.58		
II	21(72.4%)	8(27.6%)			
III	13(56.5%)	10(43.5%)			
IV	1(50.0%)	1(50.0%)			
**Immune status based on CD4 counts**					
CD4 > 350 or 20%	31	16	0.48	0.62	0.14 - 2.83
CD4 < 350 or 20%	6	5			

### Association between oral disease/ caries and intake of refined sugars

Consumption of refined sugars was at least once a day in 35 (58.4%), occasional in 14(23.3%) child, while 11(18.3%) abstained from consumption. Children who consumed refined sugars at least once a day had higher frequencies of oral soft tissue diseases amongst them. This association however lacked statistical significance (P > 0.05).

## Discussion

The 63.8% prevalence obtained for oral lesions in the present study shows that oral lesions are common among HIV infected children. Our prevalence is comparable with the prevalence of 61.9% obtained among HIV infected children from Northern Nigeria, but is much higher than the 52.6% and 41.2% prevalence estimate among Brazilian and Tanzanian HIV infected children respectively [6–8]. The differences in the geographic settings may probably be responsible for the wide disparities noticed between our result and the Brazilian and Tanzanian study. The findings of Caries and enamel hypoplasia as the most common dental lesions and candidiasis, herpes labialis and periodontal disease as the common oral soft tissue lesions in the present study is similar to the findings in the previous studies [2, 6–8]. Candidiasis was the most common oral soft tissue lesion identified and a significant reduction was recorded amongst those on HAART in the present study. The lower rates of oral lesions among those on HAART have been explained by the HAART boosted immunity among those on anti-retrovirals. Predilection of candidiasis among HIV infected children have been previously attributed to immunosuppression [2, 8]. The other common oral soft tissue lesions identified in this study include angular cheilitis, herpes labialis. These lesions were more common among children not on HAART; however this association was not statistically significant. However most of the previous studies report a statistically significant reduction of oral soft tissue lesions in HIV infected subjects on HAART [2–4]. Our findings in the present study though contrary to this are however consistent with reports from Tanzania and Thailand which document no significance for the findings of more oral soft tissue lesions amongst children not on HAART [8, 9]. Diseases of the hard dental tissues predominated amongst the oral lesions in the present study. The preponderance of hypoplasia of the enamel obtained in this study has also been reported in other studies [4, 6, 9]. The association between hypoplasia and HIV was not statistically significant in this and many of the previous studies. We presume that HIV may inhibit formation of the enamel but recommend that more studies be conducted to clarify the true position of the nature of the association between HIV infection and enamel formation. Poor oral hygiene, inadequate fluoridation and frequent exposure to refined sugars have been documented to be responsible for caries which develop when the process of tooth demineralisation is more favored than remineralisation [10, 11]. The present study however did not record a statistically significant increase among children who ingested refined sugar on a daily basis. The fluoride content of the tooth pastes and the water supplies was not researched in the present study thus it is impossible to determine its association with caries in the present study. The caregivers in the present study had poor health seeking habits because their oral health review was at the instance of this research. A low awareness on the importance of oral health may be responsible for this poor health seeking habit. Most of the care givers did not visit any dentist because they thought that they had no dental disease, yet 67.6% of those with oral lesions were not aware of the presence of disease. Regular oral health checks by qualified dentists might have identified and treated these lesions earlier. Yearly dental examinations by dentists have been associated with reduced incidence of caries in developed countries [12]. A previous report has shown that the practice of going for dental checkups is poor in Nigerian children [13]. It is thus necessary to proffer necessary strategies to bring about the positive changes to utilization of dental services in Nigeria.

## Conclusion

How can we control the problem of oral lesions in HIV infected children? More studies need to be done to identify the etiologies underlying caries and hypoplasia with a view to prevent their development. Prompt initiation of HAART is indicated for the treatment of HIV in order to prevent disease progression and further immunosuppression. Regular dental check up is also indicated for prompt detection and treatment of existing disease. Awareness on dental diseases by lectures at the clinics and public media houses may go a long way in preventing and controlling oral diseases. Dental care should also be integrated into the paediatric HIV care program.
